# Dynamic compliance in flow-controlled ventilation

**DOI:** 10.1186/s40635-021-00392-w

**Published:** 2021-05-31

**Authors:** Dietmar Enk, Julia Abram, Patrick Spraider, Tom Barnes

**Affiliations:** 1 Faculty of Medicine, University of Münster, Münster, Germany; 2 Department of Anaesthesiology and Intensive Care Medicine, Medical University of Innsbruck, Innsbruck, Austria; 3 University of Greenwich, London, UK

The recent paper by Wittenstein et al. “Comparative effects of flow- vs. volume-controlled one-lung ventilation on gas exchange and respiratory system mechanics in pigs” [1] contains some intriguing observations we would like to discuss.

## Alveolar pressure amplitude

In flow-controlled ventilation (FCV), the gas flow is constant during *both* inspiration and expiration [2, 3]. This significantly differs from volume-controlled ventilation (VCV) where inspiratory flow is constant, but exhalation is passively driven by lung–chest elasticity resulting in a decelerating flow profile. Furthermore, in contrast to VCV (and any other ventilation mode) in FCV gas is always moving either into or out of the lungs without any pause phases in an accurately controlled way. This causes a continuous pressure drop across the airway resistance. Consequently, during inspiration tracheal pressure must be higher than alveolar pressure, whereas during expiration the opposite pertains. The alveolar driving pressure (Δ*P*) in FCV is therefore lower than the measured tracheal Δ*P*.

**Fig. 1:**
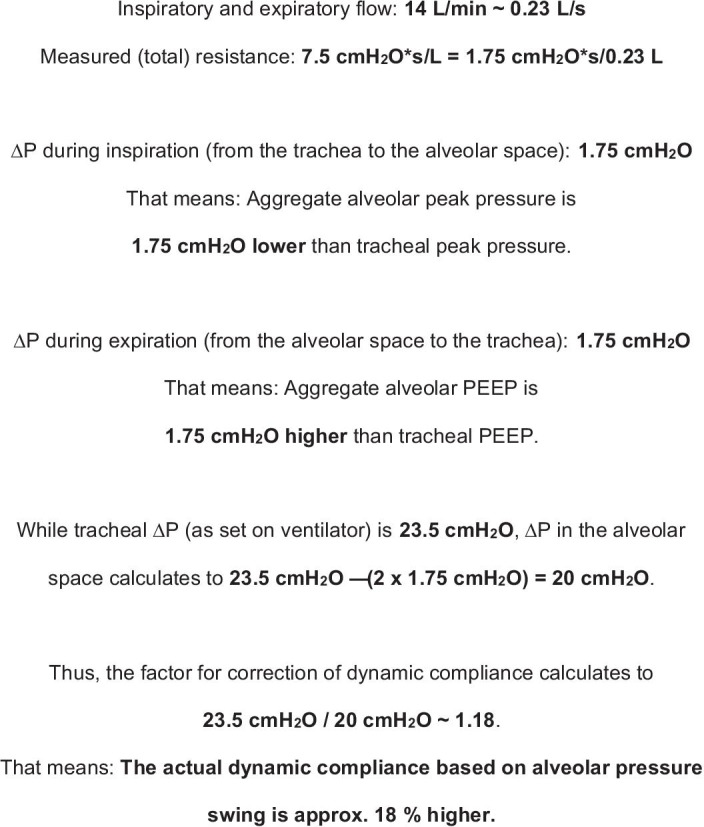
Difference between monitored tracheal pressure and aggregate alveolar pressure in flow-controlled ventilation (FCV). For calculating the dynamic compliance, the aggregate alveolar driving pressure (Δ*P*) has to be determined first by correcting the measured tracheal Δ*P* for the pressure drop across the airway resistance. Averaged data provided in [1] are used. Depending on regionally different resistances, also regional alveolar Δ*P* and dynamic compliance may be different. Because the overall lung–chest system can only be studied from the outside as a single functional unit, the calculations necessarily represent aggregate estimates of the mechanical properties of the different compartments. The calculation is based on an *I*:*E* ratio of 1:1 (which is typical for FCV) and the assumption the measured resistance is entirely related to resistance in the airways. Hence tissue resistance is not considered, so the calculated pressure drop may be slightly overestimated and, in consequence, the aggregate alveolar Δ*P* somewhat underestimated

Compliance (which necessarily means dynamic compliance considering the nature of FCV) calculated from tracheal Δ*P* will lead to an underestimation of actual (alveolar) lung compliance because tracheal Δ*P* is higher than the alveolar pressure swing. Figure 1 shows how it is possible to estimate the difference between tracheal Δ*P* and aggregate alveolar pressure swing using the measured airway resistance reported by Wittenstein et al. [1] for FCV. The calculated difference between the two pressures (partially) accounts for the difference of the compliance in both groups of the Wittenstein study. Using alveolar rather than tracheal Δ*P* for the calculation of the dynamic compliance therefore results in similar compliance in both groups.

## Dynamic compliance measurement

Gas exchange in mechanical ventilation is strongly related to dynamic compliance. If a larger volume of respiratory gas is shifted at the same compliance pressure (i.e., the part of the total pressure distending the alveolar periphery), alveolar gas exchange/turnover improves. Assuming the ventilated compartments of the lungs are perfused, so carbon dioxide is delivered, any higher alveolar gas exchange/turnover should result in increased carbon dioxide elimination. Wittenstein et al. compared FCV with VCV during one-lung ventilation (OLV) in hypovolemic and normovolemic pigs. They reported significantly better carbon dioxide elimination with FCV in normovolaemia, but substantially lower compliance—this seems counterintuitive.

Further, they reported an airway resistance of approx. 8 cmH_2_O*s/L with FCV but approx. 34 cmH_2_O*s/L with VCV—more than four times larger. Even taking into account the difference in flows (14 L/min in FCV vs. 24 L/min in VCV) and the use of a double-lumen tube with a small inner diameter as a prerequisite to perform OLV, this vast difference is surprising. In a similar double-lumen tube our own measurements show a pressure drop of 2.5 cmH_2_O at a flow of 24 L/min across the bronchial lumen (= resistance of 6.25 cmH_2_O*s/L). Obviously, this cannot explain the large difference in airway resistance between VCV and FCV.

To compare the dynamic compliance in FCV (which is calculated by the ventilator based on bronchial pressure measurements) with dynamic compliance in VCV, Wittenstein et al. had to convert the airway pressure data measured proximally of the bronchial lumen of the double-lumen tube into bronchial values using measurements of proximal airway pressure, flow, and tube Rohrer resistance. Systematic error in any of these measurements (e.g., differing flow conditions between the Rohrer resistance determination and the experiment) could cause differences between measured and actual driving pressure which may have significant effect on measured compliance, resistance, and calculated mechanical power (e.g., with a tidal volume of 220 mL at 11 mL/cmH_2_O compliance, only 2 cmH_2_O difference translates to 10% change in measured compliance). If the resistive pressure (i.e., the part of the total pressure needed to overcome resistance) is falsely high in VCV, compliance pressure amplitude will be underestimated and thus dynamic compliance (or aggregate alveolar dynamic compliance as provided for FCV in this letter) overestimated. Furthermore, the energy applied to and stored in the elastic lung tissue (i.e., elastic mechanical power) in VCV will be underestimated.

## Effects of FCV

Because of the constant flow used, FCV allows accurate measurement of relative pressure swings and delivered volumes. It offers a more individualized approach to ventilation, allowing compliance-guided setting of *both* positive end-expiratory pressure (PEEP) and peak pressure. In our experiments, it improved lung aeration homogeneity and gas exchange efficiency without detectable regional overinflation [4]. In contrast to the fixed FCV ventilator settings used in the study of Wittenstein et al., this approach may be also applicable in OLV, possibly further reducing respiratory rate and dead space ventilation while ventilating at substantially lower levels of mechanical power and dissipated energy compared to conventional ventilation modes.

## Data Availability

Not applicable.
